# Robust and reproducible population receptive field mapping in patients with retinal pathologies

**DOI:** 10.1038/s41433-026-04523-z

**Published:** 2026-06-04

**Authors:** Maximilian Pawloff, David Linhardt, Michael Woletz, Marlene Hollaus, Georgios Mylonas, Graham E. Holder, Stefan Sacu, Christian Windischberger, Markus Ritter

**Affiliations:** 1 Department of Ophthalmology, Medical University of Vienna, Vienna, Austria; 2 High Field MR Center, Center for Medical Physics and Biomedical Engineering, Medical University of Vienna, Vienna, Austria; 3 Department of Ophthalmology, Yong Loo Lin School of Medicine, National University of Singapore, Singapore, Singapore; 4 Department of Ophthalmology, National University Hospital, Singapore, Singapore

**Keywords:** Retinal diseases, Hereditary eye disease

## Abstract

**Purpose:**

Previous studies have shown high reproducibility of population receptive field (pRF) mapping in young, healthy individuals. The present study examines whether such a level of reproducibility can also be achieved in patients suffering from retinal disease.

**Methods:**

Eleven patients with Stargardt disease and eleven patients with geographic atrophy (GA) secondary to age-related macular degeneration (AMD) were examined in up to four sessions using high-resolution ultra-high field fMRI (Siemens Magnetom 7 T) and microperimetry (MP, Nidek MP-3). Reproducibility of the pRF parameters within and between sessions was assessed using Spearman’s correlation coefficient.

**Results:**

Retinotopic maps calculated from ultra-high field MRI had excellent intra- and intersession reproducibility for pRF center position (median correlation between sessions for pRF center eccentricity: *r* = 0.91; polar angle: *r* = 0.90), but only modest reproducibility for pRF size (average correlation *r* = 0.39). Reproducibility was constant across sessions multiple weeks apart, indicating a long-term stability of the method. In addition, the results show that reproducibility is not related to the severity of retinal disease.

**Conclusion:**

The data demonstrate that retinotopic mapping of the primary visual cortex using ultra-high field MRI is a highly reproducible technique for the assessment of macular function in patients with retinal disease. The technique provides an unbiased quantification of retinal function adjunct to conventional clinical assessments and may assist the early diagnosis of retinal disease. In addition, it may be a valuable objective method for monitoring visual deficits during long-term therapeutic interventions or disease progression.

## Introduction

Numerous techniques are available for the evaluation of retinal function in patients suffering from retinal disease. Subjective examination methods, such as perimetry or visual acuity testing, are most commonly used but psychophysical data may be influenced by attention levels and other subjective factors. Microperimetry (MP), for example, tests localized retinal sensitivity in the foveal, parafoveal, and peripheral macular regions [1]. Whilst MP allows for structure-function correlation and is used in clinical trials investigating new therapies, results are subject not only to patient performance and learning effects, but also to ceiling and floor effects in devices with limited luminance range [2, 3]. Although reproducibility of mean retinal sensitivity in perimetry and MP has been demonstrated in patient populations [2, 3], those studies only assessed intrasession reproducibility, with a short average interval of five minutes between tests. A separate study investigating the intersession variability of MP in patients with type 2 macular telangiectasia, where authors warranted exclusion of the first session due to statistically significant training effects and a mean test-retest variability as high as 3.3 dB, further emphasized the impact of learning effects on psychophysical measurements [4].

In contrast, functional magnetic resonance imaging (fMRI) provides an objective and non-invasive method for assessing neural activity in response to visual stimuli, independent of patient attention or subjective perception. It measures changes in the amplitude of the MR signal based on variations in neuronal activity, offering an intrinsic biological marker of functional brain responses [5]. FMRI offers the possibility to map activation patterns with high spatial resolution in cortical and subcortical areas of the brain. As such, it is the optimal tool for investigating retinotopic organization, a key feature of the human visual system. Retinotopic projection of the visual field in the visual cortex ensures that neighboring areas of the retina are encoded in adjacent cortical areas.

In retinotopic fMRI scans, patients are presented with a combination of stimuli that include rotating wedges and expanding rings or moving bars to determine eccentricity and polar angle of visual field positions in the visual cortex [6]. Population retinotopic field (pRF) mapping, an extension of this method, was developed by Dumoulin and Wandell whereby neuronal receptive fields are estimated by a model-driven approach which enables detailed representations of the visual field at the cortical level to be revealed [7]. In this model, a receptive field (RF) is described as the extent of the visual field where stimulation leads to neural activity in a specific area of the visual cortex. While it is not possible to estimate RFs of single neuron, neurons with similar receptive fields are located in close proximity to each other. This leads to the representation of a population of neurons in every cortical voxel of fMRI datasets. Hence the name of the method.

Several fMRI studies have explored cortical responses to visual stimuli in the context of retinal disease. Initial reports included a study of a patient with age-related macular degeneration (AMD) in which fMRI retinotopic mapping revealed a distinct unresponsive zone in the primary visual cortex, also known as visual area 1 (V1), corresponding to the retinal lesion [8]. A number of other studies demonstrated that fMRI-derived cortical “silent zones” closely matched perimetry-derived visual field defects in patients with retinal pathology [9–13]. Those studies used retinotopic fMRI at magnetic field strengths of up to 3 Tesla. The availability of MRI scanners operating at ultra-high fields of 7 Tesla and above has opened new possibilities as higher magnetic fields yield higher MR signal strengths and allow for higher resolution of the acquired activation maps [14]. In healthy participants, it has been shown that pRF results at 7 Tesla are highly reproducible [15] but no such data is available for clinical populations.

The present study assesses the reproducibility of pRF mapping results at 7 Tesla in patients with Stargardt disease (STGD) or geographic atrophy (GA) secondary to AMD. Both pathologies are characterized by central macular atrophy and central visual field loss. Macular lesions may initially appear perifoveally and expand with time to involve the fovea. This distinct lesion pattern makes these patients prime cases for exploring the cortical representation of central retinal scotomata. Both intrasession and intersession reproducibility were investigated.

## Methods

### Subjects

Eleven patients with genetically confirmed variants in ABCA4 (STGD; 7 male, 4 female; age: 29.3 ± 8.8 years) and eleven patients with GA (7 male, 4 female; age: 72.6 ± 5.0 years) participated. All had a secure clinical diagnosis supported by optical coherence tomography (OCT). Inclusion criteria for STGD and GA patients consisted of (1) a well-demarcated central atrophic macular lesion with or without foveal sparing, not exceeding 15° visual angle diameter and (2) fixation stability classified as stable or relatively unstable as measured by MP (see below).

The study was approved by the institutional review board of the Medical University of Vienna (EK1594/2018) and was conducted in accordance with the Declaration of Helsinki and the International Conference of Harmonization of Good Clinical Practice guidelines. Written informed consent was obtained from all patients before their participation.

### Clinical examination

Patients underwent slit-lamp examination, dilated fundus examination and best-corrected visual acuity (BCVA) testing using Early Treatment Diabetic Retinopathy Study (ETDRS) charts. A Spectralis HRA & OCT system (Heidelberg Engineering, Heidelberg, Germany) provided spectral-domain OCT (SD-OCT) and blue-light fundus autofluorescence (FAF) images. Central retinal function was assessed by microperimetry (MP-3 Nidek, Padova, Italy). Stimulus intensity ranged from 0 dB to 32 dB in 1 dB steps. The stimulus pattern consisted of a foveal 3×3 grid surrounded by three rings at a radius of 3° (8 points), 5.1° (12 points) and 7° (12 points) eccentricity. Fixation stability was assessed and classified as stable if 90% of fixations were located within a 2° circle, as relatively unstable if ≥80% of fixations were located within a 2° circle and as unstable if less than 80% of fixations were located within a 2° circle.

### Functional MRI measurements and pRF analysis

Functional MRI measurements were performed on a Siemens MAGNETOM 7 T scanner (Siemens Healthineers, Erlangen, Germany) utilizing a 32-channel head coil. Subjects participated in up to four scanning sessions, on average 23 days apart. Each MRI session lasted approximately 1 h and followed the clinical examination. The subjects completed two functional runs within each session using the CMRR EPI sequence [11] with the following parameters: isotropic spatial resolution of 1 mm, TE  =  25.2 ms, TR  =  2000 ms, field-of-view 128 × 128 mm, flip angle = 70°, GRAPPA acceleration = 2, slice spacing = 10%. Functional data comprised thirty-two slices placed perpendicular to the calcarine sulcus and covering the patients’ early visual areas. An additional functional scan with inverted phase encoding readout polarity was measured for later distortion correction using fsl topup. Within every session, a full-brain structural image was acquired using a magnetization-prepared rapid gradient-echo (MPRAGE) sequence (0.7 mm isotropic resolution, TE  =  3.66 ms, TR  =  1960 ms, flip angle = 9°, matrix size 320 ×310, field of view (FoV) = 217 × 224 mm, 224 slices) which was later used for tissue segmentation.

Subjects were able to view the back of the bore through a mirror mounted inside the head coil. Stimuli were presented on a custom-built back-projection screen attached to the patient bed. To minimize reflections in the bore, the screen was mounted as close to the subject as possible, resulting in a mean screen-to-eye distance of approximately 62 cm. The non-study eye was patched. Motion was minimized through extensive padding.

Data processing included brain tissue segmentation performed on the anatomical data using Freesurfer (https://surfer.nmr.mgh.harvard.edu). The automated segmentation result was manually corrected for topological errors. Based on these segmentation results, all further analysis was restricted to voxels located within the gray matter. Functional data was pre-processed using a custom pipeline including slice-timing, realignment, distortion correction and co-registration to the anatomical image. Spatial smoothing was applied on the volume data using an isotropic 2 mm FWHM Gaussian kernel. To reduce expected biases, the data was analyzed in volume space and projected to the surface only for visualization purposes.

Retinotopic pRF analysis was performed in MATLAB, facilitating vistasoft (github.com/vistalab/vistasoft), using the 2D Gaussian model. Independently for each voxel, the corresponding visual field location (x, y) and size of the receptive field (σ) were estimated in a two-stage fitting procedure [7]. This analysis establishes a discrete mapping between positions in the early visual cortex and locations in the visual field. The two functional runs acquired in each session were analyzed independently as well as averaged and analyzed as session average. This allows for the analysis of intra- as well as inter-session reproducibility. Based on the resulting polar angle maps the primary visual cortex was manually delineated. Analyses were restricted to V1, as it offers the most direct cortical representation of retinal input and the most reliable basis for mapping scotomata.

### Stimuli

Stimulation patterns during the fMRI scan covered the central 14° of visual angle and were presented using mrVista (Vista Lab, Stanford University, California) within the Matlab programming environment (The MathWorks, Inc., Natick, Massachusetts). The visual stimulus involved a bar moving across a screen, upon which was displayed an isoluminant reversing checkerboard pattern at a frequency of 16 reversals/sec. The bar width was 1.75°, representing 12.5% of the total stimulus area, and it traversed the screen in 18 discrete steps, each separated by 0.8° of visual angle in space, with a repetition time (TR) of 2 s. The bar moved in 8 different directions per run, and after each pass, the bar and checkerboard were rotated by 45°. Following each diagonal pass, a 12-second pause displayed a blank grey screen of similar mean luminance. Each run lasted 5 min and 36 s, corresponding to 168 volumes.

Participants were instructed to fixate on a small central dot (12 pixels or 0.22° visual angle diameter). Since accurate fixation was critical, thin diagonal lines (5 pixels or 0.09° visual angle diameter) intersecting at the fixation dot were displayed to aid patients in maintaining stable fixation. To further ensure fixation compliance and attention during the experiment, the fixation dot color changed pseudo-randomly and subjects were asked to report these changes via button press. The rate of correct detections is referred to as fixation performance.

### Reproducibility analyses

By realigning all runs within each session into the same space, direct voxel-to-voxel comparisons were possible. Before computing correlations, we applied a 20% explained-variance threshold to each run to restrict analyses to well-fitted voxels. Correlations were calculated only for voxels present in both compared datasets and runs with fewer than 300 overlapping voxels were excluded.

Reproducibility was assessed by computing Spearman’s correlation coefficient (SCC) for pRF eccentricity and size, and circular correlation coefficient [16] for polar angle across all voxels within each subject’s V1. For the intrasession comparison, the correlation was calculated between two independently analyzed runs within each session. For the intersession comparison, the two runs of each session were first averaged, and maps from session 1 were correlated with those from sessions 2–4. Sessions were excluded if fixation performance in any run fell below 50% or if the mean frame-wise displacement exceeded 1 mm.

For the comparison of mean microperimetry (MP) intensity and pRF reproducibility, each subject’s between-session pRF reproducibility values were averaged across all included sessions. MP intensity was averaged across the entire visual field and across sessions. The relationship between mean MP intensity and pRF reproducibility was then assessed across subjects using linear regression. Whenever correlation coefficients were averaged, values were first Fisher z-transformed and converted back after averaging to ensure proper statistical weighting and to correct for the nonlinearity of the correlation scale.

## Results

The reproducibility of fMRI was investigated as an objective tool for visual field-predictions in 22 patients with central visual field defects. Baseline clinical characteristics, as shown in Table 1, differed between groups. STGD patients showed worse visual acuity than GA patients (logMAR VA: STGD mean = 0.46, GA mean = 0.17; Mann–Whitney U test, *p* = 0.042). Although mean fixation stability was lower in STGD, fixation stability assessed by microperimetry did not differ significantly between groups, neither at the 2°, nor the 4° criterion (all *p* > 0.4).

**Table 1:** Baseline clinical characteristics of GA and STGD patients.

Patient Number	Sex	Age	Eye	VA logMAR	Atrophic Area mm²	% MP1 fixation 2°	% MP1 fixation 4°
**GA01**	f	73	OD	0.21	4.72	81.2	90.8
**GA02**	f	66	OS	0.03	2.18	94.8	97.5
**GA03**	m	71	OS	0.35	0.99	88.8	98.3
**GA04**	m	74	OD	0.08	23.64	93.4	99.3
**GA05**	m	68	OS	0.5	0.40	100	100
**GA06**	m	77	OS	0.14	1.96	87.9	97.9
**GA07**	f	73	OD	0.1	1.49	85.9	95.7
**GA08**	m	77	OD	0.18	21.03	88.6	98.1
**GA09**	f	64	OS	0.17	16.76	93.7	97.1
**GA10**	m	79	OD	-0.01	0.23	97.4	99.6
**GA11**	m	69	OS	0.14	2.76	82.7	95
**STGD01**	m	34	OD	0.2	2.54	99.3	99.6
**STGD02**	m	23	OD	0.84	1.29	70.6	95.4
**STGD03**	m	36	OS	0.23	0.28	94.9	96.7
**STGD04**	f	31	OS	0.91	0.53	73.5	97.2
**STGD05**	m	22	OD	1.01	2.71	48.2	90.6
**STGD06**	f	21	OD	0.5	2.33	95.7	96.6
**STGD07**	f	24	OD	0.15	2.23	99.2	99.8
**STGD08**	m	25	OD	0.2	0.20	94.5	97
**STGD09**	f	22	OS	0.12	0.28	96.6	100
**STGD10**	m	52	OS	0.06	0.42	90	96.3
**STGD11**	f	32	OS	0.82	9.15	49.5	86.5

Patient number, sex, age, examined eye, best-corrected visual acuity (VA in logMAR), OCT-derived atrophic area (mm²), and fixation stability assessed by microperimetry (MP1) using the percentage of fixation points within 2° and 4° radii are reported separately for patients with geographic atrophy (GA) and Stargardt disease (STGD).

Structural disease severity was assessed through quantifying the atrophic area using Heidelberg Engineering RegionFinder™ (Version 2.6.5.0, build: Sep. 13, 2019) on FAF images, crosschecked with Heidelberg Spectralis OCT images. Although GA patients showed a tendency towards larger atrophic areas, no statistically significant difference was observed between groups (Mann–Whitney U test, *p* = 0.212).

**Fig. 1:**
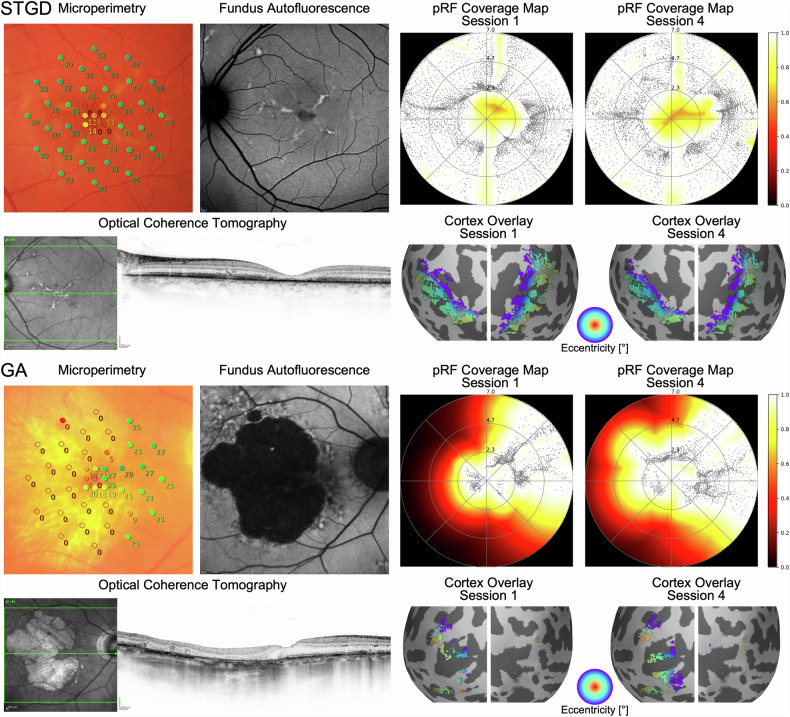
Overview of example STGD and GA patient. Comparison of Microperimetry (MP), Fundus Autofluorescence (FAF), Optical Coherence Tomography (OCT) and population receptive field (pRF) mapping results of study patients with Stargardt’s Disease (STGD) and Geographic Atrophy (GA). pRF coverage map comparison of session 1 and session 4 is shown in the top right. The coverage maps show the coverage plots where each dot corresponds to a detected pRF center, while the bottom half shows eccentricity values overlaid on the visual cortex surface.

Example patients for STGD and GA are shown in Fig. 1.

### Reproducibility

Reproducibility was assessed using 59 sessions of 17 unique subjects (9 GA, 8 STGD) after application of exclusion criteria (Six pRF sessions of GA patients and 17 pRF sessions of STGD patients were excluded due to low fixation performance, insufficient data quality or movement artifacts, resulting in 59 sessions from 17 patients to be included for analysis), by calculating Spearman’s correlation for eccentricity and pRF size and circular correlation for polar angle [16]. Intra- and intersession reproducibility were evaluated separately. Within sessions, median reproducibility was high for polar angle (GA: *r* = 0.93; STGD: *r* = 0.95) and eccentricity (GA: *r* = 0.91; STGD: *r* = 0.82), while pRF size showed lower reproducibility (GA: *r* = 0.36; STGD: *r* = 0.34). No significant group differences were observed for polar angle (*p* = 0.75) or pRF size (*p* = 0.77), whereas eccentricity reproducibility was higher in GA than in STGD (*p* = 0.008).

Between sessions, eccentricity reproducibility remained high (GA: *r* = 0.92; STGD: *r* = 0.85). Polar-angle reproducibility was substantially lower in GA than in STGD (GA: *r* = 0.74; STGD: *r* = 0.95), while pRF size reproducibility was modest in both groups (GA: *r* = 0.38; STGD: *r* = 0.42). No significant differences were found between groups for eccentricity (*p* = 0.39) or pRF size (*p* = 0.16), whereas polar-angle reproducibility was slightly lower in GA than in STGD (*p* = 0.033).

**Fig. 2:**
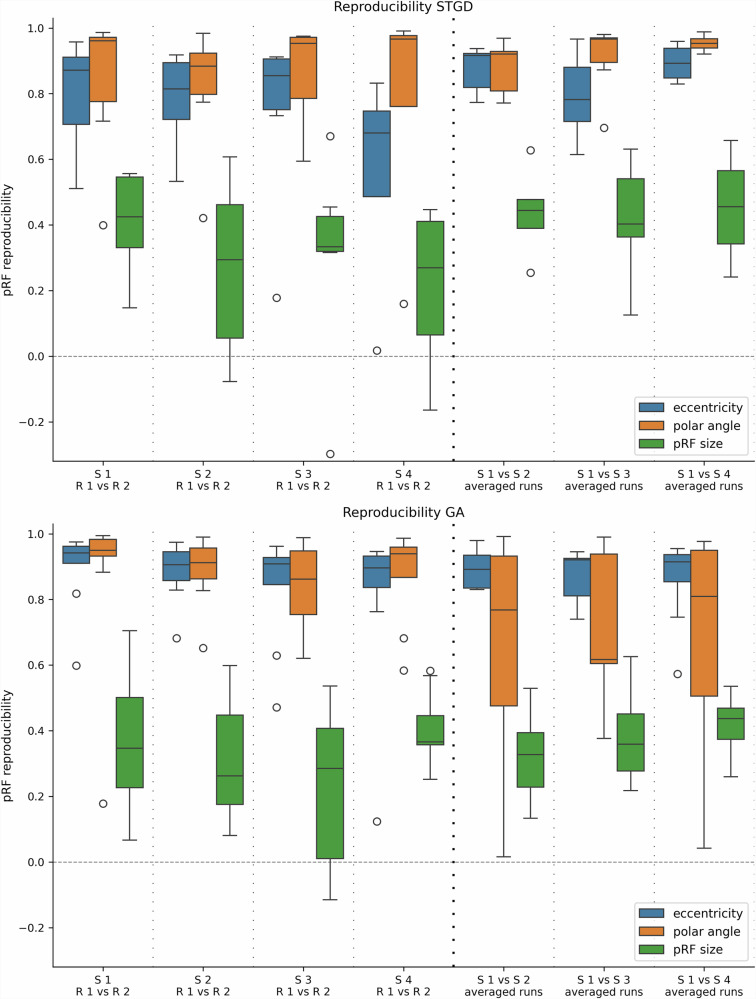
pRF reproducibility of Stargardt’s disease (STGD) and Geographic Atrophy (GA) patients. Spearman’s correlation coefficients are shown for pRF eccentricity and pRF size and circular correlation for polar angle. Columns 1–4 display intrasession comparisons, and columns 5–7 intersession comparisons. Each box represents the distribution across all included subjects within the patient group, and circles indicate identified outliers for each parameter and comparison type.

Correlation coefficients for eccentricity, polar angle, and pRF size across various intra- and intersessional comparisons are detailed in Fig. 2, with corresponding values provided in Table 2.

**Table 2:** Group-median correlation coefficients for eccentricity, polar angle, and pRF size across sessions (Ses1–Ses4) and runs (Run1–Run2).

		eccentricity	polar angle	pRF size
GA	Ses1 Run1 vs. Run2	0.94 [0.88-0.97]** p = 0.0039	0.95 [0.82-0.99]** p = 0.0039	0.35 [0.14-0.52]** p = 0.0039
	Ses2 Run1 vs. Run2	0.91 [0.80-0.96]* p = 0.0156	0.91 [0.74-0.97]* p = 0.0156	0.26 [0.06-0.44]* p = 0.0156
	S 3 Run1 vs. Run2	0.91 [0.81-0.96]** p = 0.0039	0.86 [0.64-0.95]** p = 0.0039	0.28 [0.07-0.47]* p = 0.0391
	S 4 Run1 vs. Run2	0.90 [0.76-0.96]** p = 0.0078	0.94 [0.83-0.98]** p = 0.0078	0.37 [0.25-0.47]** p = 0.0078
	Ses1 vs. Ses2 averaged runs	0.90 [0.81-0.95]** p = 0.0078	0.77 [0.20-0.95]** p = 0.0078	0.33 [0.21-0.44]** p = 0.0078
	Ses1 vs. S 3 averaged runs	0.92 [0.87-0.95]** p = 0.0039	0.62 [0.01-0.89]** p = 0.0039	0.36 [0.23-0.47]** p = 0.0039
	Ses1 vs. S 4 averaged runs	0.91 [0.83-0.96]** p = 0.0078	0.82 [0.44-0.95]** p = 0.0078	0.44 [0.36-0.51]** p = 0.0078
STGD	Ses1 Run1 vs. Run2	0.88 [0.69-0.95]* p = 0.0312	0.96 [0.82-0.99]* p = 0.0312	0.43 [0.26-0.57]* p = 0.0312
	Ses2 Run1 vs. Run2	0.81 [0.64-0.91]* p = 0.0312	0.88 [0.63-0.97]* p = 0.0312	0.30 [-0.00-0.55]ns p = 0.0938
	S 3 Run1 vs. Run2	0.86 [0.65-0.95]* p = 0.0312	0.96 [0.85-0.99]* p = 0.0312	0.33 [-0.02-0.61]ns p = 0.0625
	S 4 Run1 vs. Run2	0.68 [0.21-0.90]ns p = 0.1250	0.97 [0.60-1.00]ns p = 0.1250	0.27 [-0.08-0.57]ns p = 0.3750
	Ses1 vs. Ses2 averaged runs	0.92 [0.84-0.96]ns p = 0.0625	0.92 [0.81-0.97]ns p = 0.0625	0.44 [0.28-0.58]ns p = 0.0625
	Ses1 vs. S 3 averaged runs	0.79 [0.52-0.91]* p = 0.0312	0.97 [0.90-0.99]* p = 0.0312	0.40 [0.20-0.57]* p = 0.0312
	Ses1 vs. S 4 averaged runs	0.90 [0.78-0.96]ns p = 0.1250	0.95 [0.88-0.98]ns p = 0.1250	0.46 [0.20-0.66]ns p = 0.1250

Reported values are Spearman correlation coefficients for eccentricity and pRF size. Circular correlation coefficients were reported for polar angle. Corresponding Wilcoxon *p*-values and 95% confidence intervals are provided. Confidence intervals were computed after Fisher z-transformation and transformed back to correlation space.

**Fig. 3:**
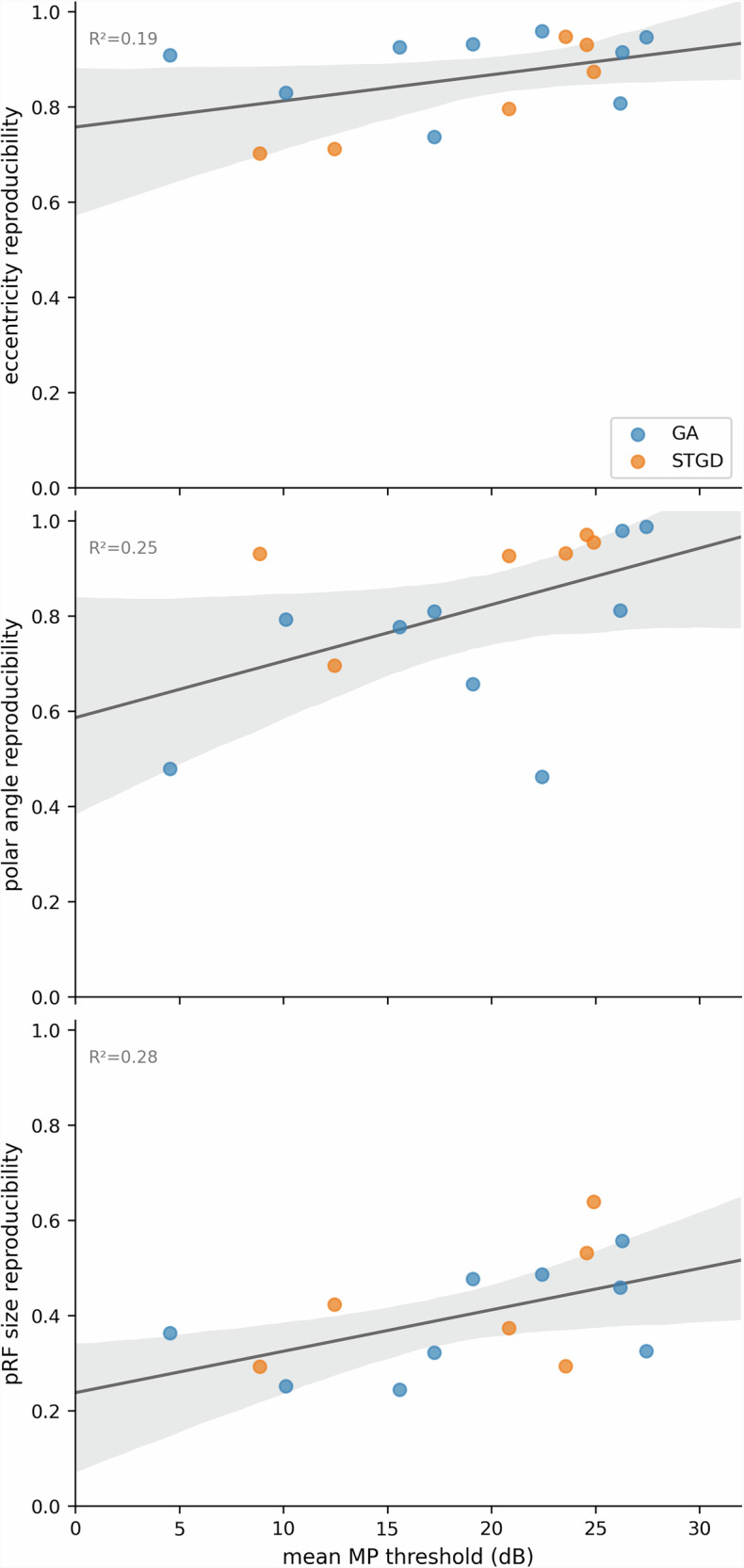
Relationship between inter-session pRF reproducibility and mean microperimetry (MP) intensity. For each subject, pRF reproducibility was computed between sessions using run-averaged maps, and both pRF reproducibility and MP intensity were averaged across the entire visual field and all available sessions. Each dot represents one subject. The grey line indicates the best-fit linear regression across subjects, with the shaded area showing the 95% confidence interval. Coefficients of determination (R²) are reported for each parameter. Explained variance is low for pRF position parameters (eccentricity and polar angle), indicating that reproducibility of pRF position is largely independent of retinal sensitivity across the observed range. Explained variance is also limited for pRF size, but modestly higher than for position parameters, consistent with a greater susceptibility of pRF size reproducibility to disease severity.

Reproducibility was further examined across the severity of retinal deficits. Mean MP values were used as measures for the extent of their respective pathology. Reproducibility of both pRF eccentricity and polar angle remained high across all scotoma sizes (see Fig. 3). The lower reproducibility of pRF size was also consistent across the range of mean MP values. Since there were no significant differences in reproducibility between visits, no training effects were detected in pRF measurements.

Across subjects, mean MP threshold showed no significant linear association with inter-session reproducibility of pRF eccentricity (Spearman ρ = 0.45, *p* = 0.092), and the corresponding regression slope was not significantly different from zero (*p* = 0.103). Similarly, polar-angle reproducibility showed a significant monotonic association with mean MP threshold (ρ = 0.67, *p* = 0.007), but the regression slope did not reach statistical significance (*p* = 0.061), indicating that the slope was not significantly different from zero for either pRF position parameter. In contrast, pRF size reproducibility exhibited a significant dependence on retinal sensitivity, with both a significant correlation (ρ = 0.56, *p* = 0.028) and a regression slope significantly different from zero (*p* = 0.044), such that higher reproducibility was observed at higher MP thresholds. Coefficients of determination ranged from R² = 0.19 to 0.28 (see Fig. 3).

To assess whether baseline clinical characteristics influenced pRF reproducibility, we examined associations between visual acuity (VA), microperimetry (MP) fixation stability within 2° visual angle, and pRF reproducibility. As described above, worse VA was associated with reduced MP fixation stability across subjects, consistent with clinical expectations. However, neither VA nor MP fixation stability showed a significant association with inter-session pRF reproducibility (VA vs. reproducibility: Spearman ρ = 0.00, *p* = 0.994; fixation stability vs. reproducibility: ρ = 0.24, *p* = 0.401; Supplementary Fig. 1). These findings indicate that baseline VA and fixation stability alone do not systematically explain reduced inter-session pRF reproducibility or the occurrence of outliers.

## Discussion

This study assesses intra- and intersession reproducibility of pRF mapping results in a clinical setting using ultra-high field MRI. Twenty-two patients suffering from either geographic atrophy (GA) or Stargardt disease (STGD) completed up to four fMRI scanning sessions. We observed high reproducibility for pRF position parameters (eccentricity, polar angle) and lower reproducibility for pRF size. Within-session reproducibility values were not substantially worse than in healthy participants without visual field loss [12, 15, 17–19] and in individual with simulated scotomata [20]. Our results underscore that even substantial deviations from the assumed smoothness of pRF centre distributions do not necessarily compromise the stability of position estimates and support the potential applicability of pRF mapping in clinical ophthalmology.

A recent study reported higher reproducibility, especially for pRF sizes due to methodological improvements [21]. The study used a logarithmically warped bar stimulus which was locally optimized to match the receptive field sizes of the corresponding region of cortical space. This approach yielded more reliable pRF estimates than a standard bar stimulus with constant width, especially for pRFs located near the central visual field (for comparison see Linhardt et al. [22], Alvarez et al. [23]). These results highlight that small adaptations in the stimulation paradigm could potentially boost pRF mapping reproducibility significantly. In future pRF mapping, stimuli could be further advanced to optimally map scotomata, thus increasing the clinical relevance of the method.

Our findings acknowledge a systematic shift of pRF centers from inside the lesion toward functional retinal regions. This shift arises from the modeling approach itself and does not necessarily reflect cortical reorganization [24]. Although explicit modeling of the scotoma, as proposed by Binda et al. [24], can partly mitigate this effect, such approaches are not feasible in patients with irregular or poorly defined scotoma borders. Moreover, recent work has shown that even when the scotoma is explicitly modeled, pRF estimates can still be biased [25], underscoring that this issue warrants further investigation. Therefore, we used a naïve modeling approach without explicit scotoma masking and focused on reproducibility rather than absolute retinotopic accuracy. Thus, the reported reproducibility reflects the stability of the pRF mapping method, including its systematic modeling biases under conditions of partial visual-field loss, rather than the reproducibility of the true retinotopic map itself.

While conventional flickering checkerboard tasks presented as full-field stimuli are much simpler than population receptive field (pRF) stimuli and have been utilized since the very early days of fMRI [26], they cannot be used for scotoma mapping on the retina, as they do not allow for mapping visual field position to visual cortex activation. For patients with unknown scotoma layout, estimation of simulated scotomata using pRF mapping, or retinotopic mapping in general, offers the opportunity simultaneously to investigate the effects of scotomata on visual cortex activation and link these effects to visual field positions. This method thus allows for a comprehensive understanding of how visual field defects affect cortical representations, providing valuable insights into the relationship between retinal pathology and cortical activation. Our analyses focused on V1 because it provides the most direct and spatially precise cortical representation of retinal input. Higher visual areas (V2–V3) are more strongly influenced by feedback and integrative processing, which may confound the interpretation of pRF changes in patients.

In this clinical setting, polar angle and eccentricity yielded very high reproducibility values (*r* = 0.90 and 0.91) on par with previously reported values in healthy cohorts (r ≥ 0.98 and >0.87) [22], whereas size showed low correlation values. Our findings further substantiate that among the parameters utilized in pRF mapping, the pRF size parameter demonstrates notably lower reproducibility [12, 15, 17–19, 21]. This underscores the need for further advancements in this technique. Notably, GA patients showed slightly lower between-session reproducibility for the polar-angle parameter compared with their within-session estimates, whereas STGD patients exhibited comparable values across sessions. This difference might reflect subtle changes in the scotoma border or local adaptation processes in GA patients, though this interpretation remains speculative.

The higher exclusion rate observed in STGD patients is likely related to baseline clinical differences between groups. Worse visual acuity and reduced fixation performance during microperimetry may go hand in hand with increased fixation instability during the pRF paradigm. This issue is particularly relevant for pRF mapping experiments, which place substantially higher demands on sustained fixation than conventional microperimetry. Consequentially, the exclusion of sessions with insufficient fixation introduces a potential selection bias, as relatively less advanced cases may have been preferentially retained for analysis. Structural disease severity was quantified using hypo-autofluorescent lesion area on fundus autofluorescence imaging. Although GA patients showed slightly larger atrophic areas on average, no statistically significant difference was observed between groups. Importantly, the high reproducibility of eccentricity and polar-angle estimates was preserved across a broad range of GA and STGD eyes, underscoring the robustness and clinical potential of ultra-high-field fMRI–based pRF mapping across a wide spectrum of disease severity.

Given the higher exclusion rate in STGD patients due to insufficient fixation performance, fixation-related bias represents an important potential confounder when interpreting pRF reproducibility. Our additional analyses show that, although poorer visual acuity is associated with reduced fixation stability as measured by microperimetry, neither baseline visual acuity nor fixation stability is systematically related to inter-session pRF reproducibility. This suggests that outliers and the reduced reproducibility of pRF size are unlikely to be solely driven by fixation-related bias or systematic instability across sessions. Instead, these effects are more plausibly explained by the inherent vulnerability of pRF size estimates to noise and session-specific variability, as discussed above. Nevertheless, the elevated exclusion rate in STGD patients introduces a selection bias toward individuals with relatively preserved fixation, which may inflate apparent stability in the retained data and should be considered when generalizing these findings.

Although patients were instructed to fixate strictly on a central fixation target and runs with insufficient task adherence were excluded, some sessions still showed apparent activation within regions of outer retinal atrophy, likely reflecting residual fixation instability. Concurrent correction of fixation errors during pRF mapping is technically challenging in ultra-high-field MRI and was not implemented in the present study. However, previous work has demonstrated that eye-tracker–based gaze monitoring and post-hoc correction can substantially improve the robustness of pRF estimates [27]. Future studies would therefore strongly benefit from the integration of standardized online eye-tracking and gaze-correction approaches in ultra-high-field (7 T) MRI, particularly when studying patient populations with impaired central vision.

Although ultra-high-field (7 T) fMRI provides a substantial increase in signal-to-noise ratio (SNR) compared with conventional 3 T scanners, this advantage did not translate into improved pRF reproducibility. In the present study, the increased SNR was primarily leveraged to achieve high spatial resolution (1 mm isotropic), which is critical for mapping small and irregular scotomata but inherently reduces gains in temporal SNR. Moreover, pRF size estimates are suspected to be particularly sensitive to physiological and microvascular noise, which is known to increase at higher field strengths.

In clinical populations, additional patient-related factors further limit the stability of pRF size estimates. Fixation instability associated with reduced visual acuity and central scotomata can impair effective stimulus sampling, stronger affecting pRF size than position parameters. Practical constraints of ultra-high-field imaging include a limited field of view, increased sensitivity to small head movements affecting both data quality and effective field coverage as well as restricted options for online eye-tracking and fixation correction. These may further diminish the net benefits of 7 T fMRI for pRF size estimation. Together, these factors suggest that while ultra-high-field imaging enables high-resolution pRF mapping, it cannot overcome the fundamental vulnerability of pRF size estimates in patients with retinal disease.

Statistical testing demonstrated that the regression slopes for eccentricity and polar-angle reproducibility were not significantly different from zero. This indicates that pRF position estimates remain robust even in patients with advanced retinal deficits. In contrast, pRF size reproducibility showed a slight but statistically significant dependence on retinal sensitivity, consistent with its generally lower stability and greater susceptibility to disease-related factors. Consequently, pRF size constitutes a more problematic parameter in clinical populations, as its reproducibility is already reduced and appears to deteriorate further in the presence of larger scotomata, limiting its reliability for longitudinal or cross-sectional interpretation.

Building on prior work demonstrating that retinotopic mapping with fMRI can serve as an objective tool to assess therapeutic responses in neovascular AMD patients undergoing anti–vascular endothelial growth factor therapy [13], the present results indicate that pRF mapping yields stable estimates in patients under clinically stable disease conditions. While this study does not address longitudinal disease progression directly, the demonstrated reproducibility supports the potential use of pRF mapping in future long-term and longitudinal studies of retinal disease. This is particularly relevant as recent studies in inherited retinal diseases have begun to explore treatment-related changes in visual processing at the level of the lateral geniculate nucleus and primary visual cortex following gene therapy, which were shown to correlate with improvements in clinical measures of visual function [28].

In conclusion, population receptive field (pRF) center parameters (polar angle and eccentricity) show high reproducibility in patients with retinal disease, whereas pRF size is substantially less stable. These findings indicate that pRF mapping can serve as a robust complement to conventional clinical assessments and support its potential use for monitoring retinal disease and evaluating therapeutic interventions, including emerging treatments such as gene or cell-based therapies.

## Supplementary information

Supplemental Figure 1.

Supplemental Figure 1 - Legend

## Data Availability

The datasets generated during and/or analysed during the current study are available from the corresponding author on reasonable request.

## References

[1] Hanout M, Horan N, Do DV. Introduction to microperimetry and its use in analysis of geographic atrophy in age-related macular degeneration. Curr Opin Ophthalmol. 2015;26:149–56. 10.1097/ICU.0000000000000153. 25784112 10.1097/ICU.0000000000000153

[2] Wu Z, Ayton LN, Guymer RH, Luu CD. Intrasession test-retest variability of microperimetry in age-related macular degeneration. Investig Ophthalmol Vis Sci. 2013;54:7378–85. 10.1167/iovs.13-12617. 24135753 10.1167/iovs.13-12617

[3] Chen FK, Patel P, Xing W, Bunce C, Egan C, Tufail A, et al. Test-retest variability of microperimetry using the Nidek MP1 in patients with macular disease. Investig Ophthalmol Vis Sci. 2009;50:3464–72. 10.1167/iovs.08-2926. 19324853 10.1167/iovs.08-2926

[4] Wong EN, De Soyza JDA, Mackey DA, Constable IJ, Chen FK. Intersession test-retest variability of microperimetry in type 2 macular telangiectasia. Transl Vis Sci Technol. Published online 2017. 10.1167/tvst.6.6.7. 10.1167/tvst.6.6.7 PMC5727942 29242756

[5] Wandell BA, Dumoulin SO, Brewer AA. Visual field maps in human cortex. *Neuron*. Published online 2007. 10.1016/j.neuron.2007.10.012. 10.1016/j.neuron.2007.10.012 17964252

[6] Sereno MI, Dale AM, Reppas JB, Kwong KK, Belliveau JW, Brady TJ, et al. Borders of multiple visual areas in humans revealed by functional magnetic resonance imaging. Science. 1995;268:889–93. 10.1126/science.7754376. 7754376 10.1126/science.7754376

[7] Dumoulin SO, Wandell BA. Population receptive field estimates in human visual cortex. Neuroimage. Published online 2008. 10.1016/j.neuroimage.2007.09.034. 10.1016/j.neuroimage.2007.09.034 PMC3073038 17977024

[8] Sunness JS, Liu T, Yantis S. Retinotopic mapping of the visual cortex using functional magnetic resonance imaging in a patient with central scotomas from atrophic macular degeneration. Ophthalmology. 2004;111:1595–8. 10.1016/j.ophtha.2003.12.050. 15288993 10.1016/j.ophtha.2003.12.050

[9] Silson EH, Aleman TS, Willett A, Serrano LW, Pearson DJ, Rauschecker AM, et al. Comparing clinical perimetry and population receptive field measures in patients with choroideremia. Investig Ophthalmol Vis Sci. Published online 2018. 10.1167/iovs.18-23929. 10.1167/iovs.18-23929 PMC6110169 29971442

[10] Carvalho J, Invernizzi A, Martins J, Jansonius NM, Renken RJ, Cornelissen FW. Visual field reconstruction using fmri-based techniques. Transl Vis Sci Technol. Published online 2021. 10.1167/tvst.10.1.25. 10.1167/tvst.10.1.25 PMC7814355 33520421

[11] Prabhakaran GT, Al-Nosairy KO, Tempelmann C, Thieme H, Hoffmann MB. Mapping Visual Field Defects With fMRI – Impact of Approach and Experimental Conditions. Front Neurosci. Published online 2021. 10.3389/fnins.2021.745886. 10.3389/fnins.2021.745886 PMC8455880 34566575

[12] van Dijk JA, de Haas B, Moutsiana C, Schwarzkopf DS. Intersession reliability of population receptive field estimates. Neuroimage. 2016;143:293–303. 10.1016/j.neuroimage.2016.09.013. 27620984 10.1016/j.neuroimage.2016.09.013 PMC5139984

[13] Ritter M, Hummer A, Ledolter AA, Holder GE, Windischberger C, Schmidt-Erfurth UM. Correspondence between retinotopic cortical mapping and conventional functional and morphological assessment of retinal disease. Br J Ophthalmol. Published online 2019. 10.1136/bjophthalmol-2017-311443. 10.1136/bjophthalmol-2017-311443 29699983

[14] Uğurbil K. Ultrahigh field and ultrahigh resolution fMRI. Curr Opin Biomed Eng. Published online 2021. 10.1016/j.cobme.2021.100288. 10.1016/j.cobme.2021.100288 PMC8112570 33987482

[15] Himmelberg MM, Kurzawski JW, Benson NC, Pelli DG, Carrasco M, Winawer J. Cross-dataset reproducibility of human retinotopic maps. Neuroimage. Published online 2021. 10.1016/j.neuroimage.2021.118609. 10.1016/j.neuroimage.2021.118609 PMC8560578 34582948

[16] Jammalamadaka SR, Sengupta A. Topics in Circular Statistics. Vol 5. World Scientific; 2001.

[17] Senden M, Reithler J, Gijsen S, Goebel R. Evaluating Population Receptive Field Estimation Frameworks in Terms of Robustness and Reproducibility. PLoS ONE. 2014;9:e114054 10.1371/journal.pone.0114054. 25463652 10.1371/journal.pone.0114054 PMC4252088

[18] Benson NC, Jamison KW, Arcaro MJ, Vu AT, Glasser MF, Coalson TS, et al. The human connectome project 7 Tesla retinotopy dataset: description and population receptive field analysis. J Vis. 2018;18:23 10.1167/18.13.23. 30593068 10.1167/18.13.23 PMC6314247

[19] Lage-Castellanos A, Valente G, Senden M, De Martino F. Investigating the Reliability of Population Receptive Field Size Estimates Using fMRI. Front Neurosci. 2020;14:825 10.3389/fnins.2020.00825. 32848580 10.3389/fnins.2020.00825 PMC7408704

[20] Linhardt D, Pawloff M, Hummer A, Woletz M, Tik M, Vasileiadi M, et al. Intra- and inter-session reproducibility of artificial scotoma pRF mapping results at ultra-high fields. J Vis. 2022;22:3471 10.1167/jov.22.14.3471. 10.1523/ENEURO.0087-22.2022 PMC9512620 36635900

[21] Chang K, Fine I, Boynton GM. Improving the reliability and accuracy of population receptive field measures using a logarithmically warped stimulus. J Vis. 2025;25:5 10.1167/jov.25.1.5. 39752175 10.1167/jov.25.1.5 PMC11702787

[22] Linhardt D, Pawloff M, Woletz M, Hummer A, Tik M, Vasileiadi M, et al. Intrasession and Intersession Reproducibility of Artificial Scotoma pRF Mapping Results at Ultra-High Fields. eNeuro. 2022;9. 10.1523/ENEURO.0087-22.2022. 10.1523/ENEURO.0087-22.2022 PMC9512620 36635900

[23] Alvarez I, de Haas B, Clark CA, Rees G, Samuel Schwarzkopf D. Comparing different stimulus configurations for population receptive field mapping in human fMRI. Front Hum Neurosci. 2015;9. 10.3389/fnhum.2015.00096. 10.3389/fnhum.2015.00096 PMC4335485 25750620

[24] Binda P, Thomas JM, Boynton GM, Fine I. Minimizing biases in estimating the reorganization of human visual areas with BOLD retinotopic mapping. J Vis. Published online 2013. 10.1167/13.7.13. 10.1167/13.7.13 PMC3689563 23788461

[25] Urale PWB, Puckett AM, York A, Arnold D, Schwarzkopf DS. Highly accurate retinotopic maps of the physiological blind spot in human visual cortex. Hum Brain Mapp. Published online 2022. 10.1002/hbm.25996. 10.1002/hbm.25996 PMC9812231 35796159

[26] Kwong KK, Belliveau JW, Chesler DA, Goldberg IE, Weisskoff RM, Poncelet BP, et al. Dynamic magnetic resonance imaging of human brain activity during primary sensory stimulation. Proc Natl Acad Sci USA. Published online 1992. 10.1073/pnas.89.12.5675. 10.1073/pnas.89.12.5675 PMC49355 1608978

[27] Hummer A, Ritter M, Tik M, et al. Eyetracker-based gaze correction for robust mapping of population receptive fields. Neuroimage. 2016;142:211–24. 10.1016/j.neuroimage.2016.07.003. 27389789 10.1016/j.neuroimage.2016.07.003

[28] Ashtari M, Bennett J, Leopold DA. Central visual pathways affected by degenerative retinal disease before and after gene therapy. Brain. 2024;147:3234–46. 38538211 10.1093/brain/awae096 PMC11370797

