# Supplementary figures and images for: Robust and reproducible population receptive field mapping in patients with retinal pathologies
Source: Eye (Lond). 2026 Jun 4;40(11):1676–85. doi: 10.1038/s41433-026-04523-z (PMC13415518; doi:10.1038/s41433-026-04523-z)

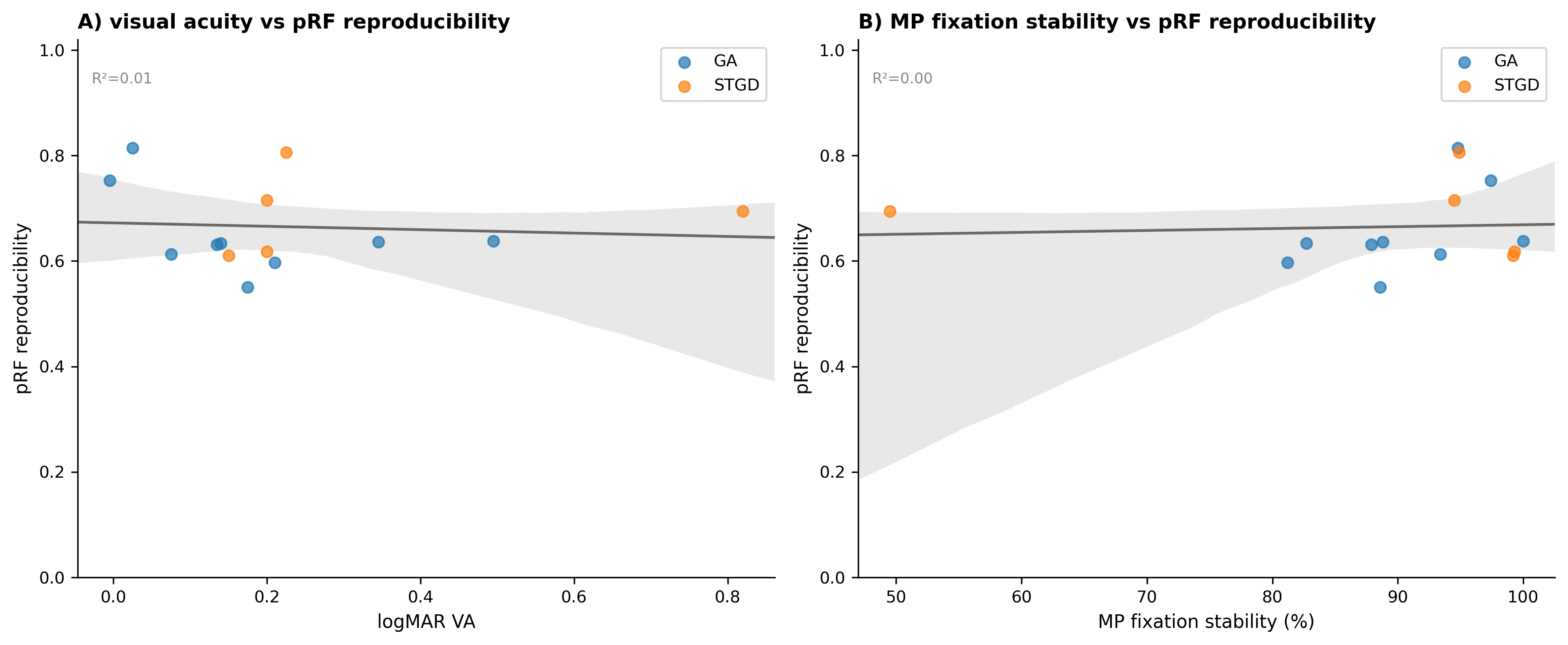

Supplement: Supplementary file 1 — Supplemental Figure 1. [file 41433_2026_4523_MOESM1_ESM.png]
