# Supplementary material for: Robust and reproducible population receptive field mapping in patients with retinal pathologies
Source: Eye (Lond). 2026 Jun 4;40(11):1676–85. doi: 10.1038/s41433-026-04523-z (PMC13415518; doi:10.1038/s41433-026-04523-z)
Supplement: Supplementary file 2 — Supplemental Figure 1 - Legend [file 41433_2026_4523_MOESM2_ESM.docx]

Supplemental File


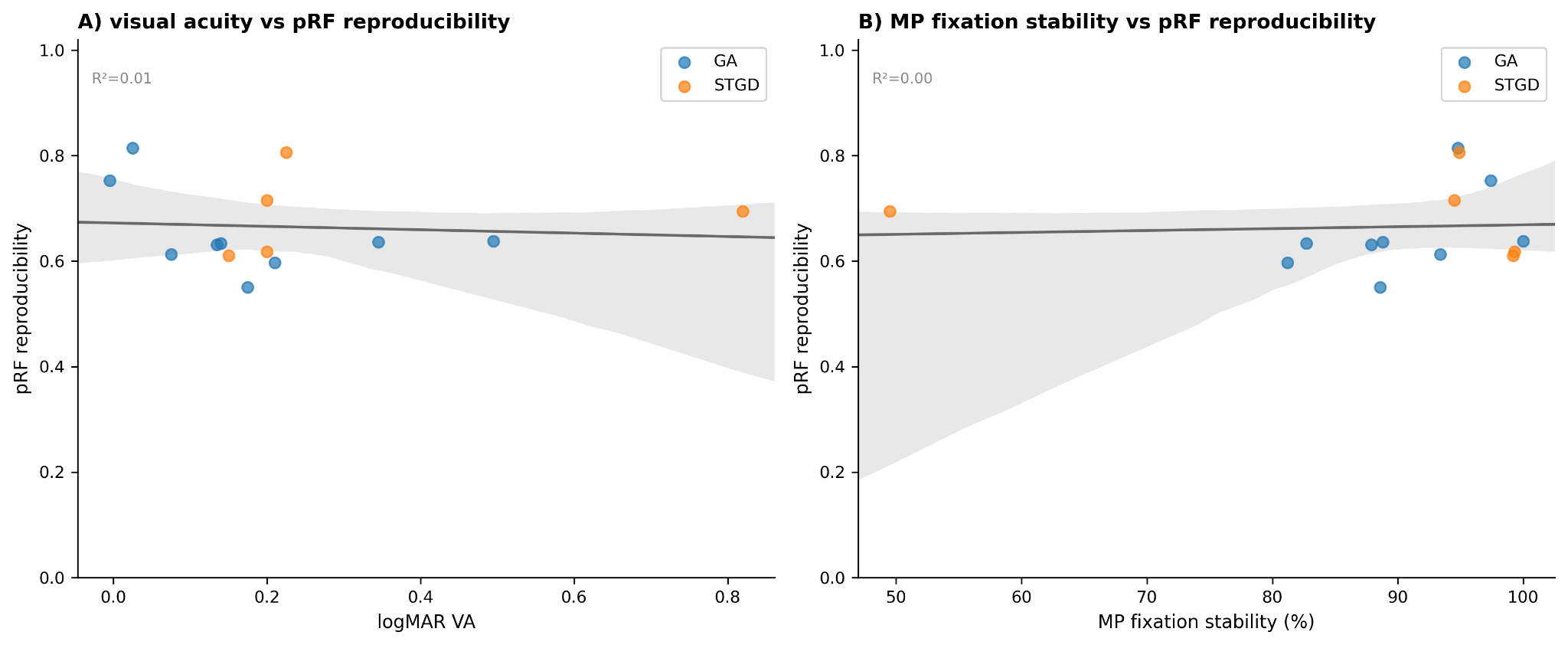


**Supplementary Figure 1.** Relationship between baseline clinical measures and inter-session pRF reproducibility. (A) Inter-session pRF reproducibility as a function of visual acuity (VA, logMAR). (B) Inter-session pRF reproducibility as a function of fixation stability assessed by microperimetry (percentage of fixation points within the predefined radius). Each dot represents one subject (GA: blue; STGD: orange). Solid lines indicate linear regression fits across subjects, with shaded areas denoting 95% confidence intervals. Coefficients of determination (R²) are reported in each panel. Neither baseline visual acuity nor fixation stability shows a systematic association with inter-session pRF reproducibility.
